# Relative deprivation and incident functional disability among older Japanese women and men: prospective cohort study

**DOI:** 10.1136/jech.2008.078642

**Published:** 2009-05-06

**Authors:** N Kondo, I Kawachi, H Hirai, K Kondo, S V Subramanian, T Hanibuchi, Z Yamagata

**Affiliations:** 1 Department of Society, Human Development, and Health, Harvard School of Public Health, Boston, USA; 2 Department of Health Sciences, Interdisciplinary Graduate School of Medicine and Engineering, University of Yamanashi, Chuo-shi, Japan; 3 Research Promotion Center for Community Care, Nihon Fukushi University, Nagoya-shi, Japan; 4 Institute of Regional Studies, Osaka University of Commerce, Higashi Osaka-shi, Japan

## Abstract

**Background:**

A prospective observational study was conducted to test the hypothesis that relative deprivation was associated with incident physical or cognitive disability, independent of absolute income.

**Methods:**

Study subjects consist of 9463 non-disabled people aged 65+ years in the Aichi Gerontological Evaluation Study (AGES), Aichi prefecture, Japan. Baseline mail-in survey in 2003 gathered information on income, educational attainment, lifestyle factors (smoking, alcohol consumption and health check-up) and healthcare utilisation. Three-year incidence of disability was assessed through public long-term care insurance databases and resident registry.

**Results:**

A total of 7673 subjects (81%) with complete information were analysed. Our measure of relative deprivation was the Yitzhaki index across eight reference groups, which calculates the deprivation suffered by each individual as a function of the aggregate income shortfall for each person relative to everyone else with higher incomes in that person’s reference group. Cox regression demonstrated that, after controlling for sociodemographic factors (including absolute income), the hazard ratio (and 95% confidence intervals) of incident physical/cognitive disability per one standard deviation increase in relative deprivation ranged from 1.13 (0.99 to 1.29) to 1.15 (1.01 to 1.31) in men and from 1.11 (0.94 to 1.31) to 1.18 (1.00 to 1.39) in women, depending on the definition of the reference group. Additional adjustment for lifestyle factors attenuated the hazard ratios to statistical non-significance.

**Conclusion:**

Relative deprivation may be a mechanism underlying the link between income inequality and disability in older age, at least among men. Lifestyle factors in part explain the association between relative deprivation and incident disability.

Population ageing in developed countries has spurred political concerns about future impacts of disability on health services utilisation, and many countries have drafted public health measures to address long-term care (LTC) prevention.^1^ For example, Japan established public LTC insurance in 2000, which places strong emphasis on individual behavioural changes.^2^ ^3^ To improve the performance of LTC prevention policies, it is also critical to understand and address the social determinants of LTC need such as income, education^4^ and income inequality.^5^ ^6^

Although still controversial, the association between income inequality and health has been examined in a wide range of countries and settings.^5^ ^6^ Two distinct hypotheses have been proposed through which income inequality is believed to affect health at the individual level. First, the absolute income hypothesis posits that an unequal society creates more people in poverty who suffer poor health due to material insufficiency.^4^ Second, the relative deprivation hypothesis posits that the degree of income inequality in society will heighten an individual’s sense of relative deprivation, resulting in frustration, shame, stress and maladaptive coping responses (such as smoking).^7^ ^8^ The theory of social comparison underlies this hypothesis.^9^ Empirical support for this hypothesis has been provided recently by studies in the Nordic countries,^10^ ^11^ the United States^12^ ^13^ and Japan,^14^ although negative studies have also been reported.^15^^–^17 However, these studies all used data in working-age populations, and evidence among the elderly remains sparse.

In this study, we sought to provide a test of the relationship between relative deprivation and the future onset of functional disability among older Japanese individuals.

## METHODS

### Study population

We used data from the Aichi Gerontological Evaluation Study (AGES), a prospective longitudinal study aimed at clarifying the role of contextual and psychosocial factors on the health and longevity of older adults. We conducted a baseline survey in November 2003 in a random sample of functionally independent 59 622 individuals aged 65 years or older residing in 15 municipalities from three prefectures in Japan. The sample was restricted to those who were not already receiving the public LTC insurance benefit. Subjects were included only if they reported no limitations in basic activities of daily living including walking, bathing and toilet use.^18^ The mail-in questionnaire also enquired about sociodemographic and lifestyle factors, that is age, gender, marital status, income, education, smoking habits, alcohol consumption, having a health check-up or not in the last few years and health status including medical care utilisation. A total of 32 891 subjects returned the questionnaire. Baseline characteristics of the participants have been reported elsewhere.^19^ ^20^ The AGES protocol was approved by the ethics committee in Research of Human Subjects at Nihon Fukushi University.

### Incident functional disability

We followed the subjects in terms of the onset of physical or cognitive disability by using the public LTC insurance database maintained by each participating municipality. We determined functional disability based on new registration in the public LTC insurance data base, that is when a person newly qualified for the insurance benefit.^21^ The qualification was based on a standardised multistep assessment of functional and cognitive impairment including a physician’s standardised examination.^2^ These criteria for determining the onset of disability have been used in previous epidemiological studies and also form the basis of health need assessment by Japanese local governments.^22^ ^23^ We also included mortality as an endpoint, certified by the local registry. As of the end of October 2006, we linked the baseline data of all 9463 participants living in the five participating municipalities in Aichi prefecture to the LTC insurance database. Of those, 7673 subjects (81%) provided complete baseline key information. People who did not provide the complete information were 26% more likely to be female, and on average 2.2 years older than those who provided the information. The LTC insurance data from the remaining 10 municipalities were not available for reasons such as delay in data handling or clerical procedures in the municipalities.

### Income and relative deprivation

The baseline survey asked about annual household pre-tax income in 2002. The income question had 14 categories, and the midpoints were set as household income in each category. The incomes for people in the top-coded category were obtained by Pareto estimation.^24^ We adjusted household income for household size, dividing the income by the square root of the number of people in that household.

Following the recently adopted method of Eibner and colleagues,^12^ ^13^ relative deprivation was operationalised in the present study using the Yitzhaki index,^25^ which is itself based on the theory of relative deprivation articulated earlier by Runciman.^26^ In brief, relative deprivation for each individual is calculated as the aggregated shortfall in income between that individual and everyone else with higher incomes in that person’s reference group:


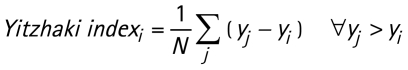


where the amount of relative deprivation for individual *i* is the sum of the income gap between individuals *i* and *j* (*y_j_* – *y_i_*, where *j* has higher income than *i*) divided by the total number of people in the reference group (*N*). As we cannot know the reference group for each individual (ie, to whom each person compares him/herself), our approach is to fit alternative definitions of reference groups: others living in the same geographical area (five municipalities), others in the same age group (65–74 or 75+ years old) or gender, others with the same educational attainment (0–9 or 9+ years of education). We also created reference groups defined by the combinations of these variables. Each reference group ranged from 1307 to 5364 subjects when a single variable was used as the basis for social comparison, and from 191 to 754 when three variables were used.

### Covariates

Other explanatory variables included age, gender, marital status (married, widowed or divorced, never married), educational attainment (less than 6 years, 6–9 years, 10–12 years or 13 years or longer) and medical care utilisation (no health problems, minor health problems not currently needing medical care, health problems needing but not currently receiving medical care due to patient choice, or currently receiving medical care). We also considered lifestyle factors including smoking history (never, ever or current), alcohol consumption (non-drinker; not drink everyday; drink 35 g of alcohol or less; or drink more than 35 g every day) and receipt of health check-up (had check-up in the last year, had it in the last 2 or 3 years, had it 4 years ago or before, or never had it).

### Statistical analysis

Cox proportional hazard models were used to calculate the hazard ratio (HR) and 95% confidence intervals (CI) for subsequent disability onset according to the level of relative deprivation. Multivariate models were adjusted for absolute income and other covariates. All models were stratified by gender.^27^ We also modelled relative deprivation further adjusted for lifestyle factors in order to assess whether such factors mediated the association between relative deprivation and the onset of disability. To address potential multicollinearity between relative deprivation and absolute income, we also carried out analysis stratified by median absolute income. In our primary analysis, both income and relative deprivation were treated as continuous variables, and income was not equivalised by household size because this approach minimised the collinearity between the two variables. We subsequently conducted a sensitivity analysis using alternative specifications of both variables (ie, continuous and quartile). The utilisation of equivalised rather than non-equivalised income as a covariate did not materially alter the results or our conclusions. All statistical analyses were performed using SAS statistical package version 9.1 (SAS Institute Inc., Cary, NC, USA).

## RESULTS

Over 11 456 person–years of observation in men and 10 216 person–years in women, we observed 191 and 286 subjects newly registered in the LTC insurance database, as well as 313 and 146 deaths respectively (total 504 and 432). The incidence rate (IR) of functional disability (ie, new registration in the LTC insurance database and mortality) was 0.044 in men and 0.042 in women. Participants who were older, unmarried or had lower educational attainment showed higher IRs. Lower income was associated with higher IR among male participants, whereas the association was unclear among women. The IR also differed by lifestyle factors. Relative deprivation was positively associated with the IR regardless of the reference groups selected for calculating the Yitzhaki index. Among men, the IR ranged from 0.031 to 0.036 in the lowest quartile and from 0.061 to 0.064 in the highest quartile; while among women, the range was from 0.043 to 0.049 in the lowest and from 0.049 to 0.053 in the highest quartile (table 1).

**Table 1:** Baseline characteristics of the subjects and incidence rate of functional disability: the Aichi Gerontological Evaluation Study (AGES), Aichi, Japan, 2003–2006

Baseline characteristics	Men (4061 respondents)		Incidence rate	Women (3612 respondents)		Incidence rate
	N	Incidence/person–year		N	Incidence/person–year	
Age (years)						
65–74	2900	234/8368	0.028	2464	157/7167	0.022
75+	1161	270/3088	0.087	1148	275/3049	0.090
Marital status						
Married	3660	420/10 371	0.040	2205	182/6357	0.029
Divorced/separated	335	69/908	0.076	1267	228/3469	0.066
Never married	22	4/58	0.069	89	19/239	0.079
Other	15	4/41	0.097	11	0/33	0.000
Household equivalised income* (ranges in million Japanese yen)						
Quartile 1 (0.88–1.57)	809	139/2216	0.063	1032	152/2887	0.053
Quartile 2 (1.58–2.46)	1118	129/3162	0.041	873	90/2494	0.036
Quartile 3 (2.47–3.17)	992	123/2806	0.044	729	66/2086	0.032
Quartile 4 (3.18–10.66)	1142	113/3272	0.035	978	124/2750	0.045
Mean (SD)			2.53 (1.35)			2.38 (1.46)
Educational attainment						
9 years or less	2225	312/6233	0.050	2213	276/6240	0.044
10+ years	1804	188/5136	0.037	1364	150/3881	0.039
Medical care utilisation						
None (no health problems)	1198	89/3478	0.026	907	68/2630	0.026
Have health problems but not have medical care by patient choice	233	26/662	0.039	238	24/682	0.035
Have regular medical care	2505	375/6962	0.054	2314	324/6467	0.050
Smoking						
Never	1104	136/3113	0.044	3317	388/9395	0.041
Ever	1900	243/5352	0.045	98	15/268	0.056
Current	944	107/2678	0.040	82	18/223	0.081
Alcohol consumption						
None	1674	286/4588	0.062	3104	392/8745	0.045
Drink 35 g/day of alcohol or less	1976	180/5685	0.032	446	31/1296	0.024
Drink >35 g alcohol/day	364	30/1054	0.028	7	2/19	0.104
Health check-up						
Yes: in last 2–3 years	2685	251/7695	0.033	2472	224/7114	0.031
Yes: 4+ years ago	597	111/1630	0.068	333	43/939	0.046
Never	684	117/1884	0.062	699	152/1850	0.082
Relative deprivation defined by geographical area (municipality) of residence (ranges ×1000)						
Quartile 1 (0–284)	1039	99/2980	0.033	871	114/2437	0.047
Quartile 2 (285–610)	1059	124/3014	0.041	835	75/2399	0.031
Quartile 3 (611–1055)	1090	135/3063	0.044	848	91/2415	0.038
Quartile 4 (1056–2579)	873	146/2400	0.061	1058	152/2965	0.051
Mean (SD)			702 (534)			813 (639)
Relative deprivation defined by age group (ranges ×1000)						
Quartile 1 (0–274)	1053	93/3035	0.031	916	112/2577	0.043
Quartile 2 (275–563)	1092	142/3076	0.046	788	79/2248	0.035
Quartile 3 (564–1023)	1042	116/2956	0.039	839	83/2403	0.035
Quartile 4 (1024–2370)	874	153/2389	0.064	1069	158/2989	0.053
Mean (SD)			705 (534)			818 (642)
Relative deprivation defined by educational attainment (ranges ×1000)						
Quartile 1 (0–292)	1007	105/2880	0.036	894	122/2490	0.049
Quartile 2 (293–631)	995	114/2828	0.040	786	67/2265	0.030
Quartile 3 (632–1031)	1149	131/3256	0.040	817	88/2334	0.038
Quartile 4 (1032–2706)	878	150/2405	0.062	1080	149/3031	0.049
Mean (SD)			696 (521)			789 (626)

*100 yen  = US$0.95 in September 2008.

Survival analysis in men indicated a statistically significant association between higher relative deprivation and incident disability across the models based on alternative reference groups (table 2). The HRs for incident disability ranged from 1.19 to 1.26 per one standard deviation (SD) increase in relative deprivation, depending on the definition of reference groups. The HRs were attenuated but still statistically significant even adjusting for absolute income, demographic factors and medical care utilisation (Model 1), except for the area/gender/age model (HR = 1.13, 95% CI 0.99 to 1.29). The adjusted HRs ranged from 1.13 to 1.15. When the models were further adjusted for lifestyle factors, the adjusted HRs were attenuated by 4–5% and were not statistically significant (Model 2). In women, in contrast, the multivariate models (Model 1) were statistically significant only in the age model (HR = 1.18, 95% CI 1.00 to 1.39, which was attenuated to statistical non-significance after additional adjustment for lifestyle factors (Model 2). In terms of absolute income, the crude HR (95% CI) per 1 SD increase was 0.84 (0.76 to 0.92) in men, and became non-significant in Model 1 ranging from 0.95 (0.82 to 1.10) to 0.98 (0.84 to 1.13). The crude HR among women was 1.02 (0.93 to 1.12) and ranged from 1.09 (0.92 to 1.28) to 1.14 (0.97 to 1.34) in Model 1.

**Table 2:** Crude and adjusted hazard ratios (95% confidence intervals) for incident functional disability per one standard deviation (SD) increase in relative deprivation: the Aichi Gerontological Evaluation Study (AGES), Aichi, Japan, 2003–2006

Reference group defined by:	Crude	Model 1	Model 2
Men			
Area	1.23 (1.13 to 1.33)	1.14 (1.00 to 1.30)	1.09 (0.95 to 1.24)
Age	1.26 (1.16 to 1.36)	1.14 (1.00 to 1.31)	1.09 (0.95 to 1.26)
Education	1.19 (1.10 to 1.30)	1.15 (1.01 to 1.31)	1.09 (0.96 to 1.25)
Area and sex	1.23 (1.14 to 1.33)	1.14 (1.00 to 1.30)	1.09 (0.95 to 1.25)
Age and sex	1.22 (1.13 to 1.33)	1.14 (1.00 to 1.31)	1.09 (0.95 to 1.25)
Education and sex	1.19 (1.10 to 1.29)	1.15 (1.01 to 1.31)	1.09 (0.96 to 1.25)
Area, sex and age	1.21 (1.12 to 1.32)	1.13 (0.99 to 1.29)	1.08 (0.94 to 1.23)
Area, sex and education	1.19 (1.10 to 1.29)	1.14 (1.01 to 1.30)	1.09 (0.95 to 1.24)
Women			
Area	1.07 (0.98 to 1.18)	1.12 (0.95 to 1.32)	1.07 (0.91 to 1.26)
Age	1.10 (1.01 to 1.21)	1.18 (1.00 to 1.39)	1.12 (0.95 to 1.33)
Education	1.07 (0.97 to 1.17)	1.16 (0.99 to 1.37)	1.11 (0.94 to 1.30)
Area and sex	1.07 (0.97 to 1.17)	1.11 (0.95 to 1.31)	1.06 (0.90 to 1.25)
Age and sex	1.14 (1.04 to 1.24)	1.18 (0.99 to 1.39)	1.12 (0.94 to 1.33)
Education and sex	1.07 (0.97 to 1.17)	1.16 (0.99 to 1.37)	1.11 (0.94 to 1.30)
Area, sex and age	1.12 (1.02 to 1.23)	1.11 (0.94 to 1.31)	1.06 (0.90 to 1.25)
Area, sex and education	1.06 (0.97 to 1.16)	1.14 (0.97 to 1.33)	1.08 (0.92 to 1.27)

Model 1 is adjusted for age, income, marital status, medical care utilisation and education. Model 2 is adjusted for the covariates in Model 1 plus smoking, alcohol consumption and health check-up. Relative deprivations are calculated using household equivalised income. Income as a covariate is not equivalised (in order to minimise multicollinearity between relative deprivation and income).

Stratified analyses by median income showed a stronger association between relative deprivation and incident disability among high-income men compared with low-income men (table 3). For example, the adjusted HRs (95% CI) per 1 SD increase in the area model were 1.19 (1.04 to 1.36) and 1.89 (1.29 to 2.79) in the low- and high-income groups respectively. Women showed no statistically significant HRs. Although the stratified models could still have non-negligible confounding of income, supplementary analysis stratified by tertiled income showed that, among men, the HRs of relative deprivation were consistently elevated with two exceptions in the middle-income strata in age and age/sex models. However, these HRs were not statistically significant, most probably because of the smaller number of incident disability cases in each stratum (Supplementary table).

**Table 3:** Adjusted hazard ratios* (95% confidence intervals) for incident functional disability per one standard deviation (SD) increase in relative deprivation by high and low absolute incomes separated by median: the Aichi Gerontological Evaluation Study (AGES), Aichi, Japan, 2003–2006 (N = 7673)

Reference group defined by:	Model 1		Model 2	
	Low income	High income	Low income	High income
Men				
Area	1.19 (1.04 to 1.36)	1.89 (1.29 to 2.79)	1.17 (1.02 to 1.33)	1.82 (1.24 to 2.68)
Age	1.21 (1.06 to 1.38)	1.76 (1.19 to 2.62)	1.19 (1.04 to 1.36)	1.71 (1.15 to 2.54)
Education	1.18 (1.04 to 1.35)	1.81 (1.25 to 2.63)	1.17 (1.02 to 1.33)	1.72 (1.19 to 2.50)
Area and sex	1.19 (1.04 to 1.36)	1.97 (1.32 to 2.95)	1.17 (1.02 to 1.34)	1.89 (1.26 to 2.83)
Age and sex	1.21 (1.06 to 1.38)	1.82 (1.19 to 2.76)	1.19 (1.04 to 1.36)	1.75 (1.15 to 2.66)
Education and sex	1.18 (1.04 to 1.35)	1.86 (1.26 to 2.72)	1.17 (1.02 to 1.33)	1.76 (1.20 to 2.58)
Area, sex and age	1.19 (1.04 to 1.36)	1.83 (1.23 to 2.71)	1.16 (1.02 to 1.33)	1.76 (1.19 to 2.62)
Area, sex and education	1.16 (1.02 to 1.32)	2.00 (1.38 to 2.89)	1.14 (1.00 to 1.30)	1.88 (1.30 to 2.72)
Women				
Area	1.07 (0.92 to 1.24)	0.83 (0.48 to 1.41)	1.05 (0.90 to 1.23)	0.89 (0.52 to 1.51)
Age	1.12 (0.96 to 1.31)	0.76 (0.45 to 1.27)	1.11 (0.95 to 1.30)	0.82 (0.49 to 1.38)
Education	1.09 (0.94 to 1.28)	0.86 (0.52 to 1.43)	1.07 (0.92 to 1.25)	0.92 (0.55 to 1.52)
Area and sex	1.06 (0.91 to 1.24)	0.84 (0.50 to 1.39)	1.05 (0.90 to 1.22)	0.90 (0.54 to 1.48)
Age and sex	1.12 (0.96 to 1.32)	0.78 (0.49 to 1.25)	1.11 (0.94 to 1.30)	0.84 (0.53 to 1.35)
Education and sex	1.09 (0.94 to 1.28)	0.85 (0.52 to 1.39)	1.07 (0.92 to 1.25)	0.91 (0.56 to 1.48)
Area, sex and age	1.06 (0.91 to 1.23)	0.88 (0.56 to 1.40)	1.04 (0.89 to 1.22)	0.95 (0.60 to 1.50)
Area, sex and education	1.06 (0.91 to 1.23)	1.00 (0.62 to 1.61)	1.04 (0.89 to 1.21)	1.05 (0.66 to 1.68)

Model 1 is adjusted for age, marital status, medical care and education. Model 2 is adjusted for the covariates in Model 1 plus smoking, alcohol consumption and health check-up.

In sensitivity analyses using alternative specifications of income (table 4), the adjusted HRs per 1 SD increment of relative deprivation (in the education model, adjusted for income, demographic factors and medical care) were generally higher than 1.13 whatever the specification of income variable (table 4). The adjusted HRs by relative deprivation increased across increasing quartiles. Variance inflation factors were the lowest in the primary analysis (where both income and relative deprivation were continuous).

**Table 4:** Sensitivity analysis by alternative specifications of relative deprivation and income: adjusted HRs (95% CI) for incident functional disability in men: the Aichi Gerontological Evaluation Study (AGES), Aichi, Japan, 2003–2006

	Variable specification (relative deprivation/income)								
	Continuous/continuous (primary analysis)		Continuous/quartile		Quartile/continuous		Quartile/quartile		
	HR (95% CI)	VIF	HR (95% CI)	VIF	HR (95% CI)	VIF	HR (95% CI)	VIF	
Relative deprivation defined by education									
Continuous	1.15 (1.01 to 1.31)	2.66	1.13 (0.96 to 1.33)	4.14					
Quartile 1 (least deprived)					Reference		Reference		
Quartile 2					1.12 (0.78 to 1.59)	2.67	0.96 (0.68 to 1.34)		2.39
Quartile 3					1.27 (0.83 to 1.93)	4.27	1.10 (0.73 to 1.64)		4.06
Quartile 4					1.66 (1.02 to 2.72)	5.07	1.55 (0.94 to 2.54)		5.66
Absolute income	0.98 (0.84 to 1.13)	2.69							
Continuous					1.01 (0.84 to 1.21)	3.95			
Quartile 1			1.18 (0.70 to 2.00)	4.96			1.06 (0.63 to 1.80)		4.77
Quartile 2			1.11 (0.78 to 1.57)	2.85			1.08 (0.70 to 1.65)		3.83
Quartile 3			1.24 (0.96 to 1.60)	1.86			1.29 (0.93 to 1.79)		2.87
Quartile 4 (most affluent)			Reference				Reference		
Relative deprivation defined by area of residence, sex and age									
Continuous	1.13 (0.99 to 1.29)	2.70	1.10 (0.93 to 1.30)	4.47					
Quartile 1 (least deprived)					Reference		Reference		
Quartile 2					1.23 (0.88 to 1.71)	2.48	1.13 (0.82 to 1.54)		2.20
Quartile 3					1.29 (0.89 to 1.87)	3.43	1.20 (0.84 to 1.70)		3.15
Quartile 4					1.55 (0.99 to 2.42)	4.33	1.34 (0.86 to 2.08)		4.59
Absolute income									
Continuous	0.95 (0.82 to 1.10)	2.59			0.97 (0.83 to 1.14)	3.16			
Quartile 1			1.33 (0.79 to 2.23)	5.08			1.34 (0.84 to 2.13)		3.78
Quartile 2			1.17 (0.82 to 1.65)	2.82			1.13 (0.78 to 1.63)		3.03
Quartile 3			1.29 (1.00 to 1.67)	1.82			1.25 (0.93 to 1.66)		2.31
Quartile 4 (most affluent)			Reference				Reference		

VIF, variance inflation factor; HR, hazard ratio; CI, confidence intervals. HRs of continuous variables are per one standard deviation increase. All models are adjusted for age, marital status, medical care utilisation and education. Two models with the largest and smallest effect sizes of relative deprivation are selected for sensitivity analysis. VIFs higher than 10 are commonly considered to be problematic.

## DISCUSSION

Our 3-year prospective study is consistent with the relative deprivation hypothesis in male though not in female Japanese elderly. Our findings are consistent with studies from Nordic countries and the United States, as well as our previous study in Japan.^10^^–^14 Eibner and Evans found the associations between relative deprivation (measured by the Yitzhaki index) and increased mortality, smoking and obesity in a cohort of over 500 000 US working-aged men.^12^ Another US study reported an association between the Yitzhaki index and increased mental health service utilisation.^13^ We also found a significant cross-sectional association between the Yitzhaki index and poor self-rated health in a Japanese national sample.^14^ A Swedish study found an association between relative deprivation and poor self-rated health using an alternative approach in the evaluation of relative deprivation—to be relatively deprived was defined as having an income below 70% of the mean income level in the reference group.^10^ Data from three Nordic countries showed that the high relative income position in the country of residence was strongly associated with lower odds of long-standing illness.^11^

On the other hand, recent studies in Britain only partially support the relative deprivation hypothesis. Jones and Wildman used 11-year panels from British Household Panel Survey and reported mixed results across models using the Yitzhaki index.^16^ Using the same sample, Lorgelly and Lindley also failed to corroborate the hypothesis.^17^ Gravelle and Sutton found very weak association between relative deprivation and poor self-assessed health among men in British General Household Survey samples.^15^ However, the study by Jones and Wildman indicated a positive result when modelling with ordinary least squares (OLS) but not in other approaches (ie, fixed-effect, random-effect and Houseman–Taylor approaches).^16^ Therefore, the discrepancies among studies might be due to the differences in modelling approaches including covariance adjustment and hierarchical modelling.

Mental disorders (eg, depression) are important consequences of psychological stress. Eibner and colleagues demonstrated a significant link between relative deprivation and mental disorder.^13^ Many studies have reported that depression predicts functional decline.^22^ ^28^^–^32 Increased psychosocial stress due to relative deprivation may make older people vulnerable to depression and subsequent disability. In the present study, lifestyle factors in part explained the positive association between relative deprivation and disability onset. Psychological distress attributed to relative deprivation may lead to maladaptive health behaviours, thereby leading to early onset of functional disability.

We found no significant association between relative deprivation and disability among women. As a potential explanation, household income may not be a good measure capturing the relative socioeconomic position of Japanese female elderly who are likely to be economically inactive at this stage in the life course and not the main earner in their households. That is, income might not be an important gauge when people compare themselves with others. Alternatively, our sample in women could be more biased given the higher rate of missing data among females.

**What is already known on this subject**

Although income inequality is hypothesised to be a threat to population health, few studies have addressed the mechanisms through which income inequality adversely affects population health. Relative deprivation is one such pathway.

**What this study adds**

- Using a large prospective database, this study suggests that relative deprivation in income is associated with incident functional disability independent of absolute income among older Japanese men but not among women. Lifestyle factors in part explained men’s excess risk for disability according to relative deprivation.
- Men with higher income may be more vulnerable to relative deprivation than those with lower income in Japan.
- The potential implication of this study is that disability prevention should go beyond the individual level and address broader issues of income distribution in Japanese society.

The association between relative deprivation and incident disability was stronger among men with higher incomes, suggesting that the population is more sensitive to psychosocial stress arising from invidious comparisons. The same pattern was found in two studies in Nordic countries suggesting that psychosocial mechanisms could be more important than material mechanisms in a relatively egalitarian society (such as Nordic countries and Japan) where equality is a common value.^10^ ^11^ Because wealthier individuals do not suffer material deprivation in such nations, psychosocial mechanisms may be the primary pathway linking income inequality and health among the population. However, this is not in agreement with a study in the USA (where inequality is high) showing significant association between relative deprivation and mortality.^12^

This study has limitations. First, older individuals might make comparisons that were not captured in this study, such as with other people’s lifestyles portrayed in the national media. Second, relative deprivation is collinear with income.^15^ However, the consistent results in our sensitivity analysis (table 4) support the validity of our primary results. Third, we did not account for any temporal changes in time-varying variables. For example, absolute income and relative deprivation can change through the follow-up period, which could affect the participants’ health. However, our study subjects’ incomes are probably relatively stable, as pensions account for the majority of their incomes. Fourth, relative deprivation may be a marker of unobserved characteristics that predict both income (and hence relative position in the income hierarchy) as well as health status. Reverse causation is also possible, that is, unhealthy people may become relatively poor in their reference groups. Fifth, our endpoint could be biased. Because the qualification process for obtaining LTC insurance benefit requires voluntary application, individuals’ social support might alter their personal decision to apply for the benefit. The severity of functional disability (other than mortality cases) can vary ranging from those requiring only partial care in activities of daily living to being completely bedridden. Last, we did not directly evaluate psychological health outcomes, which might be a more direct test of the psychosocial impact of relative deprivation.

In sum, the present study suggests that the psychosocial effect of relative deprivation on incident disability in older ages is independent of absolute income.^6^ Our findings have potential implications for disability prevention policies in that they suggest going beyond focusing on disability prevention at the individual level, and addressing broader issues of income distribution in Japanese society.
